# A cytoplasmic pathway for gapmer antisense oligonucleotide-mediated gene silencing in mammalian cells

**DOI:** 10.1093/nar/gkv964

**Published:** 2015-10-03

**Authors:** Daniela Castanotto, Min Lin, Claudia Kowolik, LiAnn Wang, Xiao-Qin Ren, Harris S. Soifer, Troels Koch, Bo Rode Hansen, Henrik Oerum, Brian Armstrong, Zhigang Wang, Paul Bauer, John Rossi, C.A. Stein

**Affiliations:** 1 Department of Medical Oncology, City of Hope, 1500 East Duarte Road, Duarte CA 91010, USA; 2 Department of Cancer Immunotherapeutics and Tumor Immunology, City of Hope, 1500 East Duarte Road, Duarte CA 91010, USA; 3 Department of Molecular Medicine, City of Hope, 1500 East Duarte Road, Duarte CA 91010, USA; 4 Pfizer Research Technology Center, 620 Memorial Drive, Cambridge, MA 02139, USA; 5 bioTheranostics, 9640 Towne Center Dr., Suite 100, San Diego, CA 92121, USA; 6 Roche, Fremtidsvej 3, Horsholm, DK 2970, Denmark; 7 Department of Neuroscience, City of Hope, 1500 East Duarte Road, Duarte CA 91010, USA; 8 Department of Molecular and Cellular Biology, City of Hope, 1500 East Duarte Road, Duarte CA 91010, USA

## Abstract

Antisense oligonucleotides (ASOs) are known to trigger mRNA degradation in the nucleus via an RNase H-dependent mechanism. We have now identified a putative cytoplasmic mechanism through which ASO gapmers silence their targets when transfected or delivered gymnotically (i.e. in the absence of any transfection reagent). We have shown that the ASO gapmers can interact with the Ago-2 PAZ domain and can localize into GW-182 mRNA-degradation bodies (GW-bodies). The degradation products of the targeted mRNA, however, are not generated by Ago-2-directed cleavage. The apparent identification of a cytoplasmic pathway complements the previously known nuclear activity of ASOs and concurrently suggests that nuclear localization is not an absolute requirement for gene silencing.

## INTRODUCTION

Chemically modified antisense oligonucleotides (ASOs) can potentially be just as or even more effective than siRNAs and can be designed to modulate or silence the expression of virtually any cellular gene (1,2). As such, these molecules have vast applicability for research and clinical purposes. Phosphorothioate (PS) ASOs have most commonly been employed experimentally in antisense-based strategies (3). Newer generations of chemical modifications (e.g. 2′-MOE, LNA (4,5)) have produced PS ASOs that are more stable to nucleases and generally less toxic than first generation unmodified PS DNA ASOs. Modifications are often made to each nucleotide constituent of the ASO chain (6). These fully-modified ASOs are highly resistant to endonuclease activity and can be employed as steric blockers to modulate translation, splicing and non-sense mediated decay (2). However, to elicit mRNA degradation by RNase H, a gap of 6 or greater unmodified DNA nucleotides must be incorporated into the design of the ASO (7). Here, the addition of locked nucleic acid (LNA) modifications has several advantages, such as increased stability against nucleases, increased binding affinity with mRNA targets, and the preservation of RNase H recruitment to the DNA/RNA duplex (7). 16 nucleotide LNA PS-ASO gapmers with two or three LNA moieties placed at the 3′ and 5′ molecular termini (8) have been successfully used for these purposes (7).

We have previously demonstrated that PS-ASOs can be internalized by cells *in vitro* without the use of any transfection reagents; this can result in targeted gene silencing for as many as 240 consecutive days (9). We named this process ‘gymnosis’ (9,10). Gymnosis is a productive silencing pathway that has been subsequently described by others as ‘free uptake’ (11). However, ‘free uptake’ actually refers to an older observation that phosphorothioate oligos can enter cells without transfection reagents. Before the discovery of gymnosis, there was no realization that the oligos were functional. In contrast to what has been commonly been observed after the lipofection or microinjection of ASOs (i.e. significant nuclear accumulation of the ASO (12–14)), gymnotic delivery results predominately in cytoplasmic ASO distribution (9,15). The literature describing intracellular ASO localization has indicated that ASOs that elicit RNAse H activity can bind and degrade their targeted RNA in the nucleus (16,17) where the vast majority of the intracellular RNase H is localized (18). However, in the absence of transfection reagents, ASOs taken up by cells have been detected predominantly in the cytoplasm [(19) and this work]. It is not known if RNase H ASOs can degrade targets in the cytoplasm.

In this work, we investigated the intracellular localization and antisense silencing ability of RNase H-activating 3′ and 5′-LNA-modified gapmer PS-ASOs (PSL-ASOs) targeted to the Bcl-2 mRNA. Confocal microscopy showed that the intracellular distribution of the ASO heavily favored GW-degradation bodies in the cytoplasm; only a small amount of ASO was present in the nuclear compartment. The silencing of Ago-2, a protein instrumental in localizing siRNA to GW-bodies, yielded a significant loss in ASO silencing ability. In addition, the gymnotically-delivered or transfected PSL-ASO silencing of a cytoplasmic target, a 5′-end capped, 3′-polyadenylated mRNA delivered by lipo-transfection, demonstrated that ASOs seem to direct mRNA degradation in the cell cytoplasm. This novel demonstration of a putative cytoplasmic oligonucleotide-directed mRNA degradation pathway shows that nuclear localization is not an absolute requirement for antisense-mediated gene silencing. These findings may have significant ramifications in the design of ASOs for therapeutic applications demonstrating that gapmers can be also used to silence targets that are mostly or exclusively cytoplasmic (e.g. the fact that RNA can be actively degraded in the cytoplasm by gymnotically delivered ASOs could be critical for novel anti-viral therapy strategies).

## MATERIALS AND METHODS

### Plasmid constructs and cells culture conditions

The Rab7:GFP fusion gene was polymerase chain reaction (PCR) amplified from the homologous plasmid (donated by Dr Yujun Wang) using the following primers: 5′-ATTTCCGGTGAATTCATGAGCAAGGGCGAGGAAC and 5′-CGCGGCCGCTCTAGATCAGCAACTGCAGCTTTCC. The PCR product was directly cloned into pLVX-EF1a-puro (Clontech) using the Cold Fusion Cloning Kit (SBI).

For most of the experiments, HT 1080 cells, were grown in DMEM (Media Tech/Cell gro) supplemented with 10% fetal calf serum (Gemini) and 1 mM L-glutamine. Cells were seeded at ≈50% confluency 24 h prior to treatment with varying concentrations of PSL-ASOs, as indicated in the text, and then further incubated for 48 h. At the end of this time, the cells were lysed with RIPA buffer and the lysates collected for analysis. All ASOs, except where otherwise specified, were delivered to cells via gymnosis (10). The sequence of the anti-Bcl-2 PSL-ASO (SPC2996) is given in (10). The scrambled oligo sequences used as controls are also described in (10). For the eGFPmRNA experiments the anti-eGFP oligo sequence was 5′ GAACTTCAGGGTCAGC 3′ and for the ErbB3 experiments the control scrambled sequence was 5′CGCAGATTAGAAACCT 3′ with three LNA modifications at each end.

In this work, all oligos are all-PS with LNAs and at the 3′ and 5′ termini. Experiments performed to determine whether two or three LNAs at each terminus were optimal gave identical results.

### Plasmids and siRNA transfection

HT 1080 cells were seeded at 50% confluency in DMEM containing 10% FBS 24 h prior to transfection. Five μg of plasmid per 6 well dish were transfected using Lipofectamine^®^ 2000 (Life Technology) or XFect (Clontech) as recommended by the manufacturer. XFect or Lipofectamine^®^ 3000 (Life Technology) were used exclusively when double treatments were performed. The siRNAs were delivered at a final concentration of 20–50 nM using TransIT^®^ siQUEST (Mirus Bio LLC) through a reverse transfection procedure as recommended by the manufacturer. Gene silencing was achieved by transfecting the specific siRNA/shRNA or by employing the corresponding stably lentivirally-transduced line expressing a shRNA; both silencing procedures resulted in the identical outcome. For Western analysis, all transfections were performed in 6 well dishes. After ≈32 h of incubation, cells were lifted and reseeded in 12 or 24 well plates for gymnotic delivery of the PSL-ASOs. On the next day, cells were lysed in RIPA buffer and the lysates were collected for analysis.

The 5′-capped, 3′-polyadenylated eGFP mRNA (abbreviated eGFP-mRNA; TriLink Bio Technologies) transfection was performed in a 96-well plate. HT1080, HeLa or HCT-116 cells were seeded at ≈30% confluency. Approximately 18 h later, 0.5 μM or 1 μM (both concentrations yielded identical outcomes) of an anti-GFP PSL-ASO or a scrambled PSL-ASO control were added to the media. Forty-eight hours later, the media was aspirated, the cells were washed once with PBS and then transfected with 200 ng of eGFP mRNA/well using Lipofectamine^®^ 2000 (Life Technology). Fluorescence images were acquired the following day with a Nikon microscope.

Cy5-labeled, 5′-capped, 3′-polyadenylated eGFP-mRNA (abbreviated Cy5 eGFP-mRNA) was directly transfected or mixed with unlabeled eGFP-mRNA at a ratio of 4:1 or 3:2 [40 ng (or 30 ng) of Cy5 eGFP mRNA and 10 ng (or 20 ng) of eGFP-mRNA] to increase eGFP protein detection, since the Cy5 label reduces translation efficiency. The addition of unlabeled mRNA did not change the ratio of Cy5 mRNA fluorescent signal or eGFP protein expression between the controls and the corresponding experimental samples under any of the experimental conditions. These experiments were performed in both HT1080 or HeLa cell lines and yielded identical results. The anti-GFP PSL-ASO or the non-targeting PSL-ASO control were delivered via gymnosis at a 1 μM concentration. Twenty-four hours later, 30 nM of either these ASOs or 30 nM of an eGFP-targeting siRNA were transfected in additional wells using Lipofectamine^®^ 3000 (Life Technology) as recommended by the manufacturer. Twenty-four hours after transfection (48 h after gymnosis), 50ng of eGFP mRNA were delivered to each well using MessengerMax^TM^ (Life Technology) as recommended by the manufacturer. The RNA and protein expression levels were monitored at 5, 12 and 24 h after transfection. The experiments were repeated three times in duplicate wells for each treatment. For each experimental sample, fluorescence was quantified over entire wells containing equivalent number of cells. Image-Pro^®^ Premier was used for all measurements and calculations.

### Microscope imaging

The HT 1080 parental cells, the stably lentiviral transduced Rab7:egf expressing cells and cells reverse-transfected (using Lipofectamine^®^ 2000 (Life Technology)) with 400 ng of Rab7:RFP expression plasmid and either 400 ng of Dcpb1:GFP or 400 ng of GW182:GFP expression plasmids were directly seeded in 12 well glass-bottom plates (MatTeck Corporation) at ≈50% confluency. The following day, the media was replaced and 250 nM of a 5′-Cy5 labeled PSL-ASO or a lipo-complex (TransIT^®^ siQUEST) containing 50 nM of Cy5-labeled siRNA (targeted to the Stat-3 mRNA) were added to each dish. The following day, Z-section images were acquired using a Zeiss confocal microscope.

To visualize the Golgi or the endoplasmic reticulum (ER), HT 1080 cells were seeded in 6-well glass bottom plates (MatTeck) at 50% confluency. The following a 5′-Cy5-labeled PSL-ASO was added to the media (final concentration 0.75 μM). Twenty-four hours later the Golgi and the ER were stained by washing the cells once with PBS and adding a PBS solution of 3μM BODIPY FL C5-Ceramide (Life Technologies) or 1 μM of ER-Tracker green (Life Technologies) respectively, followed by 25–30 incubation at 37°C. Nuclei were stained with Hoechst 33342 (Thermo Scientific Pierce).

Lysotracker^®^ (Life Technology) was added to cells treated with 250 nM of the Cy5-labeled PSL-ASO 2 h prior to imaging to visualize lysosomes, as recommended by the manufacturer.

### Preparation of lysates for co-immunoprecipitation

Cells were grown in complete medium to a confluency of 80–90% in a 10 cm dish. The media was removed and the cells were washed twice with 5 ml cold PBS. One ml of cold lysis buffer (50 mM Tris ph 8.0, 5 mM EDTA, 150 mM NaCl, 0.1% NP-40) with 1X protease inhibitors (Complete Mini 1 836 153, Roche Diagnostics) and 50 units of RNase inhibitor (RNasin N2111, Promega) were added to the plate. Plates were kept in ice while the cells were scraped and collected into microfuge tubes. Samples were incubated in ice for 30 min and then frozen in liquid nitrogen. The samples were subsequently thawed in cool water and the cell debris was collected by centrifuging samples in a microfuge tube at 4°C at top speed for 5 min. Protein concentrations were determined using the Bio-Rad protein assay system (Bio-Rad Laboratory).

### Co-immunoprecipitation

Stable cell lines expressing FLAG-tagged Ago-1, 2, 3 or 4 or FLAG alone (Mock) were seeded at 50% confluency in 10 cm dishes. Approximately 18 h later, 50 nM of PSL-ASO was added to the medium for gymnotic delivery. Twenty-four hours later, cells were lysed as described above. One to two mg of lysate protein was used for each co-immunoprecipitation. The volume was adjusted to 1 ml with 1X protease inhibitors, 50 units/ml RNase inhibitor and 50 μl of anti-FLAG M2 beads (Sigma F 3165). After overnight incubation at 4°C, the beads were pelleted by centrifugation at 4°C at 8000 rpm for 1 min. The pellets were washed five times with buffer (50 mM Tris, pH 8.0, 5 mM EDTA, 150 mM NaCl) for 10 min at 4°C followed by a 2 m centrifugation at 3000 rpm. After the final wash, the immuno-precipitated complex was released from the beads by adding 25 μl of 3X FLAG cleavage peptide (Sigma), according to the manufacturer's instructions. The supernatants were collected by a 2 min centrifugation at 3000 rpm, fractionated in 7M urea-8% PAGE and transferred onto Hybond-N+ membrane (Amersham-Pharmacia Biotech). ^32^P-radiolabeled 21mer probes complementary to the endogenous mir16 guide sequence or a ^32^P-radiolabeled 16mer complementary to the PSL-ASO were used for the hybridization reactions, which were performed for 16 h at 37°C. A ^32^P-radiolabeled 25mer probe complementary to a sequence present in the U6 small nuclear RNA was used as control. The sequences of all probes are available upon request.

### Immunoblotting

Proteins from cell lysates were resolved by 10% SDS-PAGE. The monoclonal mouse anti-human Bcl-2 antibody (Dako) was added at 1000X dilution in TBST containing 5% fatty acid free BSA (Sigma). The monoclonal mouse anti-human α-tubulin antibody (Sigma) was added at 4000X dilution in TBST containing 5% fat-free dry milk. The secondary ECL anti- Mouse IgG-HRP (GE Health care) whole antibody was added at a 1:7000 dilution.

### Ago2/PAZ Biacore analysis

Human Ago-2 PAZ domain (Ala227 to Ser371) was over-expressed as a His_6_-tagged construct in *Escherichia coli* BL-21(DE3) and purified with its His tags enzymatically (thrombin) removed (20). Binding studies (direct and competition) were carried out using a Biacore S51. All experiments were performed at 25°C in 1X HBS running buffer (10 mM HEPES, 150 mM NaCl, pH7.4 with 3 mM EDTA, 1 mM TCEP (Sigma) and 0.005% NP20) with a flow rate of 90 μl/min. A 3′-end sense-strand biotinylated 21-mer ds siRNA (sense strand sequence: 5′-AGGAGAUGGAGAAAGGGCUUU-3′) was first immobilized to Biacore SA sensor chips at a surface protein level of ∼ 1000 RU via the biotin/streptavidin interaction. Surface activities of the siRNA were validated by running unlabeled Ago-2 PAZ domain at 25 nM. Mixtures of 25 nM Ago2 PAZ domain and 250 or 500 nM of the siRNA reference were injected over the surfaces at 110s association time and 600s dissociation time. Two Biacore sensorgrams were collected for each oligo competitor (PS- or PO- 5′-TCTCCCAGCGTGCGCCAT) and at each oligo concentration. The experiments were repeated at least two times.

### 5′ RNA ligase-mediated RACE

Total RNA was isolated from 518A2 or LNCaP cells by direct lysis in TRIzol (Invitrogen) following gymnosis. Total RNA was measured for purity using a Nanodrop spectrophotometer and RNA quality was confirmed by gel electrophoresis (1% agarose in Tris-borate buffer). 5′ RNA ligase–mediated–RACE (5′ RLM RACE) was performed according to the Invitrogen GeneRacer manual with modifications. Primers for the Bcl-2 and androgen receptor (AR) targets were designed using PrimerQuest software (http://www.idtdna.com/Scitools/Applications/Primerquest/). 5 μg of total RNA was mixed with 650 ng of the GeneRacer RNA adaptor (5′- CGACUGGAGCACGAGGACACUGACAUGGACUGAAGGAGUAGAAA-3′), heated to 65°C for 5 min and snap-cooled on ice prior to ligation. RNA ligation was performed at 37°C for 1 h in 1X ligase buffer, 20 U RNaseOut (Invitrogen) and 25 U RNA ligase (Ambion, Inc.), and then purified by phenol/chloroform extraction and ethanol precipitation. 10 μl of the RNA ligation product (2.5 ug) was reverse transcribed using SuperScript III (Invitrogen) and a Bcl-2-specific reverse primer (5′-tgcaggtgccggttcaggtactcag-3′) that was chosen to hybridize to a target site 3′ to the predicted Ago-2-mediated mRNA cut site. Reverse transcription was carried out at 55°C for 50 min followed by heat inactivation at 70°C for 15 min and snap cooling on ice. PCR was performed using 1 μl of the cDNA in a reaction with forward (GeneRacer 5′ primer: 5′-GCACGAGGACACTGACATGGACTGA-3′) and Bcl-2-specific reverse primer (5′-CATCCACAGGGCGATGTTGTCCACC-3′) which hybridizes to the target upstream of the specific primer used for the reverse transcription reaction. PCR was performed using touchdown PCR conditions of 94°C for 2 min (1 cycle), 94°C for 30 s and 72°C for 1 min (5 cycles), 94°C for 30 s and 70°C for 1 min (5 cycles), 94°C for 30 s, 65°C for 30 s and 68°C for 1 min (25 cycles) and 68°C for 10 min (1 cycle) according the GeneRacer protocol. A second round of 25 cycles nested PCR was performed using 2 μl of the reaction product from the first PCR step in a reaction with forward (GeneRacer Nested: 5′-GGACACTGACATGGACTGAAGGAGTA-3′) and a Bcl-2-specific nested reverse primer (5′-AGCTGGCTGGACATCTCGGCGAA-3′). PCR reactions were run on a 2% TBE Agarose 1000 (Invitrogen) gel, stained with 0.5 μg/ml ethidium bromide, and correctly-sized PCR products were excised from the gel and cloned into the pSC-A-amp-kana vector according to the manufacturers’ instructions (Stratagene). The identity of PCR products was confirmed by sequencing using a vector-specific T3 primer. A similar primer design strategy and RT-PCR conditions were used to amplify the cleaved AR mRNA product using the following AR-specific primers: AR RT primer (5′-CGACTGCGGCTGTGAAGGTTGCTGT-3′); AR PCR primer (5′-TCATCCAGGACCAGGTAGCCTGTGGG-3′); AR nested PCR primer (5′-AGCGTGCGCGAAGTGATCCAGAACC-3′).

### Real time PCR analysis

Cells were seeded in 6-well plates at 50% confluency in DMEM (Media Tech/Cell grow) containing 10% FBS (Gemini), 24 h prior to PSL-ASO treatment. Following an additional 24 h, total RNA was isolated by direct lysis in TRIzol (Invitrogen). One microgram of RNA per sample was reverse transcribed using a random primer and M-MLV (BioChain) Reverse Transcriptase according to the manufacturer's protocol. Twenty to fifty nanograms of the resulting cDNA were used for qPCR amplification. The qPCR reaction was performed for 40 cycles with Power SYBR green PCR master mix (ABI) at an annealing temperature of 60°C for 1 min. The hBcl2 qPCR forward primer (5′-GGATGCCTTTGTGGAACTGT-3′) and the hBcl2 qPCR reverse primer (5′-AGCCTGCAGCTTTGTTTC-3′) were used at a final concentration of 100 nM. For the ErB3 analysis, cells were seeded in 6-well plates at 50% confluency in DMEM (Media Tech/Cell grow containing 10% tetracycline-free FBS (Clontech) 24 h prior pretreatment with doxycycline (5μg/ml). Cells were incubated for an additional 48 h. The media was then replaced and 2.5 μM of ErB3 oligo (5′ TAGCCTGTCACTTCTC 3′ with three LNA modifications at each end) was added. Six hours later the doxycycline was reintroduced and cells were collected after an additional 48 h incubation. The experiment was carried out in parallel with doxycycline untreated cells. Total RNA was isolated by direct lysis in TRIzol (Invitrogen). ErB3 mRNA levels were determined by quantitative real-time PCR (qRT-PCR) using the QuantiTect Probe RT-PCR kit (Cat#: 204443; Qiagen) according to the manufacturer's instructions. The sequences for the primers were: 5′TGCAGTGGATTCGAGAAGTG-3′ and 5′-GGCAAACTTCCCATCGTAGA-3′. The sequence of the probe was 5′-CATTGCCCAACCTCCGCGTG-3′. The mRNA was normalized to the endogenous glyceraldehyde 3-phosphate dehydrogenase (GAPDH) mRNA, which served as an internal control. The primers used for the GAPDH were 5′- CCACCCAGAAGACTGTGGAT-3′ and 5′- TTCAGCTCAGGGATGACCTT-3′ and the probe was 5′- ACTGGCGCTGCCAAGGCTGT-3′. ErB3 mRNA levels were determined from the calculated threshold cycle using the iCycler iQ Real-time Detection System software. Each sample was analyzed in triplicate.

## RESULTS

### PSL-ASO localization after gymnotic delivery mirrors the intracellular localization of transfected siRNAs

It has been known for years that PS-ASOs transfected with lipid-based reagents rapidly localize to the cell nucleus (13), where their silencing activity is believed to depend on RNase H cleavage of the mRNA target after the DNA:RNA duplex has been formed (21). When a PS-ASO is gymnotically (i.e. without any transfection vehicle) delivered to cells in tissue culture, the ASO enters the cell via a combination of adsorptive and fluid-phase endocytosis (22). Thus, it follows a more ‘natural’ endocytotic pathway, one that is independent of the effects of the transfection vehicle either on the endosomal membrane or on other cellular pathways (23).

**Figure 1.**
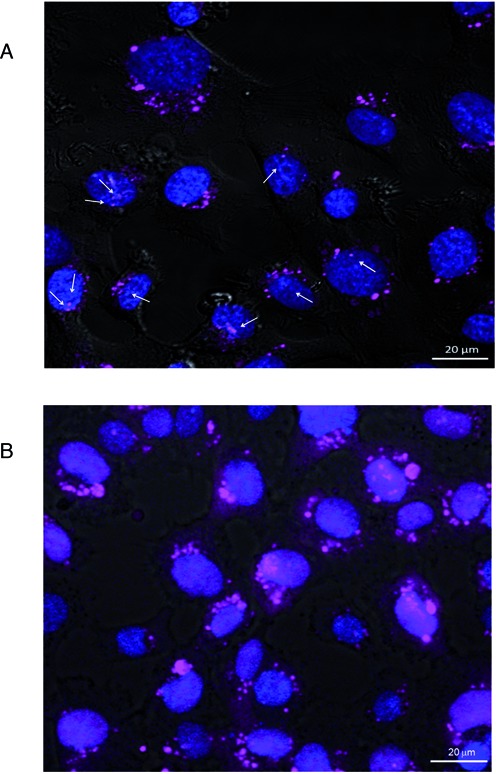
Antisense oligonucleotides (ASO) preferentially localize to the perinuclear region of the cytoplasm. (**A**) Confocal Z-section imaging of a Cy5-labeled anti Bcl-2 PSL-ASO (magenta) of HT-1080 cells demonstrates perinuclear accumulation of the fluorescent signal. Arrows indicate nuclear localization. (**B**) Confocal Z-section imaging of the same Cy5-labeled PSL-ASO delivered by lipid-assisted transfection, shows increased nuclear localization (purple). For nuclear staining, Hoechst 33342 was added at 1 μg/ml for 5 min at 37°C.

**Figure 2.**
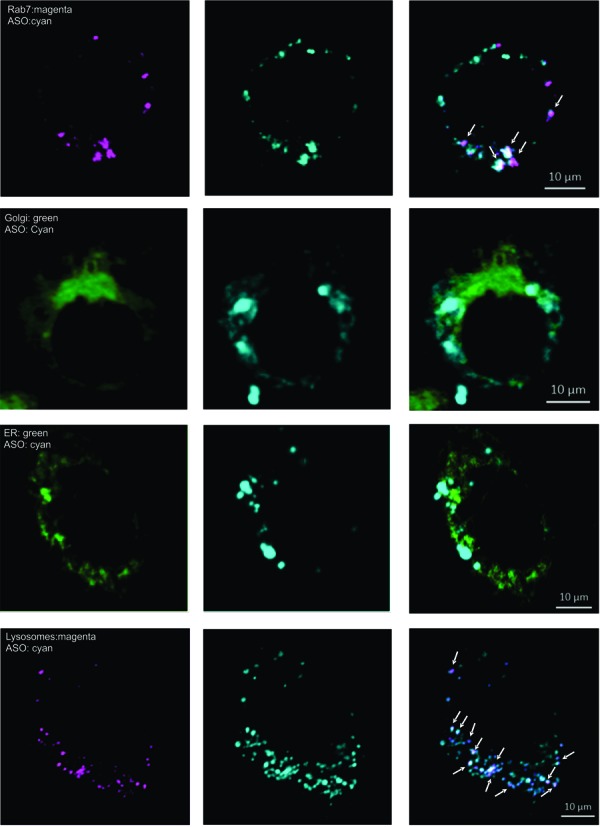
Cytoplasmic distribution of PSL-ASO. Confocal Z-section imaging of a Cy5-labeled anti-Bcl-2 PSL-ASO (Bcl-2-ASO, shown in cyan) in the perinuclear region of HT-1080 cells. The co-localization of the anti-Bcl-2 PSL-ASO and various cellular bodies is shown in the images in the far right column. The top row indicates partial co-localization of the anti-Bcl-2 PSL-ASO (cyan) and Rab7 (magenta); a marker for late endosomes. Co-localization appears as a white-pink color. The second row demonstrates the absence of or minimal co-localization between the Golgi (green) and the anti-Bcl-2 PSL-ASO (cyan), despite the oligo being in proximity to the Golgi. The third row indicates the absence of or minimal co-localization of the antiBcl-2 PSL-ASO (cyan) and the endoplasmic reticulum (ER, green). The fourth row indicates partial colocalization of the anti-Bcl-2 PSL-ASO (cyan) and the lysosomes (magenta); co-localization appears as a white-pink color.

Confocal micrographs demonstrate that the limited amount of PSL-ASO entering the nuclear compartment after gymnotic delivery forms small, discrete nuclear bodies (as indicated by the white arrows in Figure 1A), confirming previous observations (24,25). However, in contrast to lipofected PSL-ASOs, which effectively localize in the nuclear compartment (Figure 1B), the majority of the gymnotically delivered PSL-ASO localizes in the perinuclear region of the cytoplasm, where it forms additional distinct bodies (Figure 1A). Since the preferred entry route of gymnotically delivered PS oligos is a combination of adsorptive and fluid phase endocytosis (22), we used a marker for late endosomes, Rab-7, fused to red fluorescent protein (RFP), to label the late endosomes. As expected, we found the PSL-ASO in late endosomes (Figure 2, top row). However, significant amounts of ASO were also distributed outside these vesicles. Endosomes can transfer their cargo to the Golgi apparatus and to the endoplasmic reticulum (ER). They can also recycle their cargo to the cell surface or fuse with lysosomes (26). Staining for the Golgi or the ER (Figure 2, middle rows) demonstrated that the PSL-ASO was in close proximity to these cellular structures, but no apparent co-localization was detected. In contrast, the PSL-ASO could be seen in lysosomes 48 h after gymnotic delivery (Figure 2, bottom row), which is consistent with a previous observation in hepatocytes (15).

**Figure 3.**
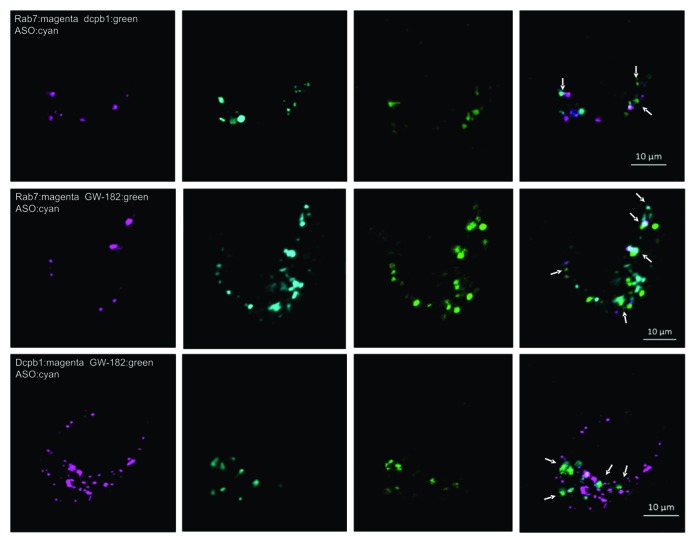
Anti-Bcl-2 PSL-ASO distribution in degradation bodies. The top row indicates partial localization of the Bcl-2-ASO (cyan) with Rab7 (magenta), as in Figure 2, and with dcpb1 (green), a marker for p-bodies. Co-localization of the Bcl-2-ASO with p-bodies is indicated by arrows. The second row indicates partial Bcl-2-ASO localization with Rab7 (magenta) and GW-182 bodies (green). Co-localization of the PSL-ASO and the GW-182 is indicated by the arrows. The third row indicates that the Bcl-2-ASO predominantly co-localizes with GW-182 bodies, as indicated by the arrows. Dcpb1 is shown in magenta, the GW-182 bodies in green and the Bcl-2-ASO in cyan. This pattern is similar to the intracellular location of siRNAs (Supplementary Figure S1).

In recent years, a distinction has been made between the GW-bodies and the p-bodies, which were previously believed to be the same entity (27). Though the GW- and p-bodies share some key proteins, their relative expression differs between the two bodies. We employed an RFP- or GFP-dcp-b1 fusion protein construct as a marker for p-bodies, and a GFP-GW-182 fusion protein construct as a marker for GW-bodies. Simultaneous marking of late endosomes using a Rab7:RFP fusion protein and p-bodies using a dcp-b1:GFP fusion protein demonstrated partial co-localization of the PSL-ASO in both compartments (Figure 3, top row). Marking the endosomes and the GW-bodies using a GW-182:GFP fusion protein also demonstrated co-localization of the PSL-ASO with these degradation bodies (Figure 3, middle row). To determine if the ASO preferentially co-localized to either p-bodies or GW-bodies, we simultaneously identified both structures using dcp-b1:RFP and GW-185:GFP fusion protein constructs. Confocal microscopic analysis indicated that a substantial proportion of the PSL-ASO co-localized with GW-bodies (Figure 3, bottom row). Remarkably, this is the identical pattern to that seen when a siRNA is delivered to cells by lipids (Supplementary Figure S1), or by a dendrimer or by silica particles (not shown).

### Ago-2 modulates PSL-ASO function

**Figure 4.**
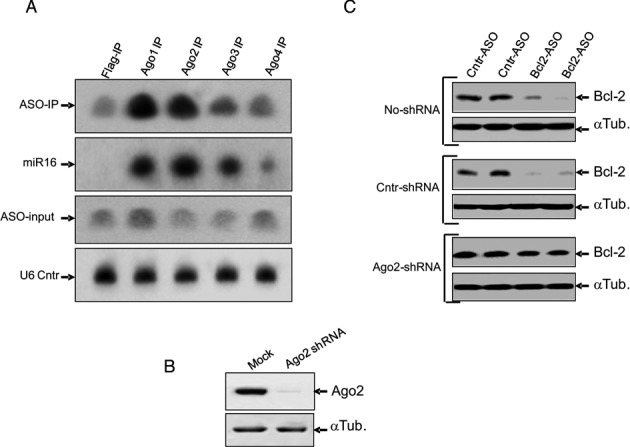
Ago-2 binds PSL-ASOs and contributes to their function. (**A**) Immuno-precipitation (IP) of Argonaute complexes followed by nucleic acid recovery and gel analysis. A P32-labeled probe fully complementary to the PSL-ASOs sequence (as described in the Experimental Procedures) was used to detect its presence in the immuno-precipitates. A P32-labeled oligonucleotide complementary to the mature sequence of miR16 was used as a control for the immuno-precipitation of the Argonaute complexes. The U6 small nucleolar RNA was employed as a loading control. The initial PSL-ASO input is also shown. (**B**) Western analysis demonstrates specific downregulation of Ago-2 by a U6-driven anti Ago-2 shRNA (25,32). A scrambled U6-shRNA was the control for specificity (Mock); an antibody to α-tubulin (α-Tub) was the loading control. (**C**) Western analysis of lysates harvested from cells treated with an anti-Bcl-2 PSL-ASO (Bcl-2-ASO) following Ago-2 downregulation. The PSL-ASOs specifically and effectively silences Bcl-2 gene expression in non-transfected cells (No-shRNA) and control cells (Cntr-shRNA) transfected with a scrambled shRNA control. However, PSL-ASOs induced gene silencing is impaired in cells with decreased levels of Ago- 2 (Ago2-shRNA; see b). A scrambled PSL-ASO (Cntr-ASO) was the control for the specificity of Bcl-2 PSLASO silencing, while α-tubulin (α-Tub) was the loading control. Each sample is shown in technical duplicates; however, the experiment was performed at two different time points and with two different PSL-ASOs concentrations. The results were analogous (not shown).

Based on the results shown above, we postulated that the PSL-ASO could progress from localization in late endosomes to localization in GW-bodies in a manner similar to siRNAs (27). This process could occur through an interaction with Ago-2, which in turn interacts with the GW-182 protein (28). To evaluate this possibility, we employed HEK-293 stable cell lines expressing either FLAG-tagged Ago 1, 2, 3 or 4 (29) and delivered a PSL-ASO via gymnosis. We then performed an immuno-precipitation (IP) with an anti-FLAG antibody, followed by detection with a ^32^P-labeled anti-ASO probe (Figure 4A). This experiment, which demonstrated an interaction of the PSL-ASO with Argonaute complexes, was reproducible when the FLAG-tagged Argonautes were transiently transfected in HT 1080 cells (Supplementary Figure S2). An anti-Bcl-2 (in HT1080 cells) or anti-androgen receptor (AR) PSL-ASO (in HEK-293 cells) were used to perform the immuno-precipitation experiments (Supplementary Figure S2 and Figure 4A). To determine if this interaction is biologically significant, we delivered an anti-Ago-2 shRNA by lipofection and silenced the intracellular expression of Ago-2 (Figure 4B). We then examined the ability of the anti-Bcl-2 PSL-ASO, after gymnotic delivery, to silence Bcl-2 protein expression following depletion of Ago-2 (Figure 4C). Experiments were performed in HT1080 wild type cells (Figure 4C, no-shRNA), in HT1080 cells treated with a control shRNA (Figure 4C, Cntr-shRNA) and in HT1080 cells treated with the Ago-2 directed shRNA (Figure 4C, Ago2-shRNA). A non-targeting PSL-ASO (Cntr-ASO) was used as a control to exclude non-specific effects. While the anti-Bcl-2 PSL-ASO could effectively silence Bcl-2 protein expression following gymnotic delivery to the HT 1080 parental (Figure 4C, top panel) or the HT 1080 control (Figure 4C, middle panel) cells, the ASO could only partially silence its target following reduction of Ago-2 expression (Figure 4C, bottom panel). We obtained essentially identical results using a tet-inducible shRNA directed against a different region of the Ago-2 transcript (29). The ASO-targeted gene for this experiment was the ErbB3 mRNA (Supplementary Figure S3).

**Figure 5.**
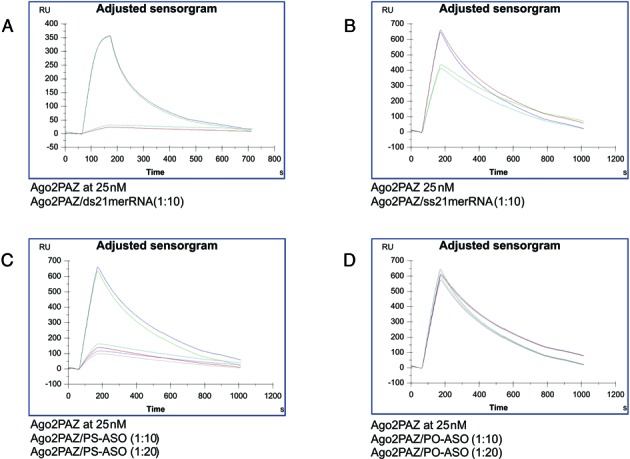
PSL-ASOs bind to the Ago-2 PAZ domain. A reference siRNA was immobilized to BiacoreR SA sensor chips and mixtures of 25 nM Ago2 PAZ domain and 250 or 500 nM of the siRNA reference or the modified oligonucleotides (the anti-Bcl-2 PS-ASO and the anti-Bcl-2 PO-ASO sequences are given in the Experimental Procedures) were injected over the surfaces. Two Biacore sensorgrams were collected for each concentration and the experiments were repeated at least two times.

To verify that the outcome of these experiments was due to a direct interaction of the PSL-ASO with Ago-2, we performed a Biacore^R^ competition experiment employing a biotinylated siRNA reference captured via the streptavidin interaction on a sensor chip. The Ago-2 PAZ domain alone or mixed with either a double stranded siRNA (ds-siRNA), a single stranded siRNA (ss-siRNA), an anti-Bcl-2 PS-ASO, or an anti-Bcl-2 PO-ASO at Ago-2 PAZ:oligo molar ratios of 1:10 and/or 1:20, was injected over the chip surface (Figure 5). Under these experimental conditions, any Ago-2 PAZ bound in solution to these putative competitors will not bind to the immobilized siRNA. As expected, the Ago-2 PAZ domain no longer bound to the immobilized siRNA surface when ds-siRNA was in the mixture (Figure 5A), while the presence of ss-siRNA produced only a minimal reduction of Ago-2 PAZ domain binding to the immobilized siRNA surface (Figure 5B). The PS-ASO was demonstrated to be a strong competitor of siRNA binding to the Ago-2 PAZ domain as it nearly abolished Ago-2 PAZ domain binding to the immobilized siRNA surface (Figure 5C). In contrast, we observed no reduction in Ago-2 PAZ domain binding to the immobilized siRNA surface when a PO-ASO was added to the mixture (Figure 5D), indicating that the PO-ASO was a very weak competitor at the concentrations we employed.

**Figure 6.**
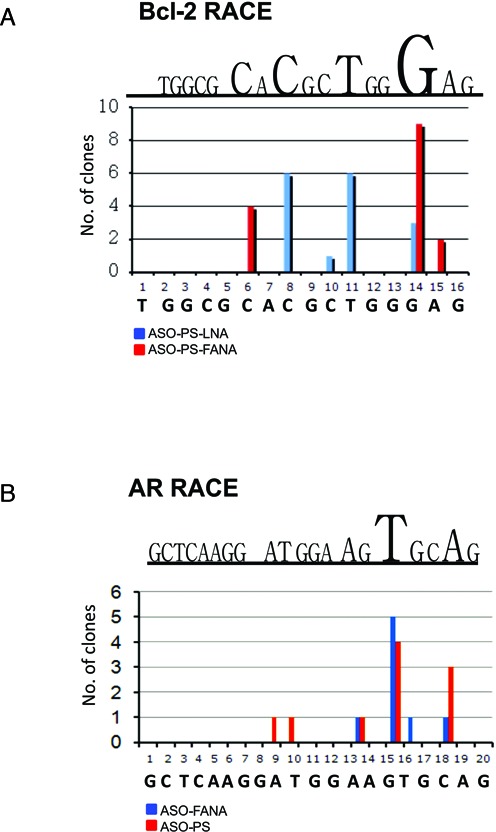
Race PCR demonstrates that Ago-2 does not direct cleavage of a target mRNA. The terminal nucleotides of the various ASO-directed mRNA cleavage products were mapped by 5′ RACEPCR. Partial sequences for the Androgen Receptor (AR) and the Bcl-2 mRNAs are shown. The Y-axes indicate the number of cloned PCR products terminating at specific nucleotides. In addition to the PSL-ASO the experiment was carried out with the 2′-fluoro-4′-thioarabino (2′ F-ANA) modification of the same oligos. The uncovered cleavage pattern is not entirely typical of RNase H (28), and could be generated by other nucleases.

To further investigate the biological significance of the binding of a PS-ASO to the PAZ domain of Ago-2, we performed a RACE PCR assay on two different mRNA targets. In both cases, the cleavage patterns of the PSL-ASO- (anti-Bcl-2 and anti-AR) targeted mRNAs were not typical of the single cleavage site characteristic of Ago-2 (Figure 6). An anti-Bcl 2 siRNA or an anti-survivin siRNA were run in parallel as controls for the RACE PCR assay and displayed the expected single cleavage site at nucleotide 10–11 (not shown). These data indicate a role for Ago-2 which differs from the catalytic function it exerts during RNA interference (RNAi).

### A PSL-ASO can target and degrade its complementary mRNA in the cell cytoplasm

**Figure 7.**
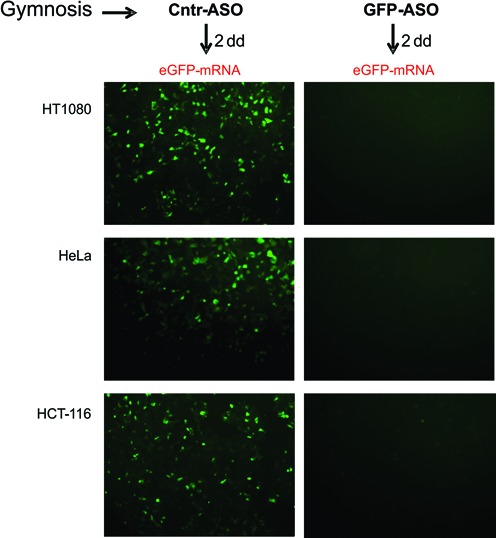
A PSL-ASO can target and degrade mRNA in the cell cytoplasm. An oligo targeted against the eGFP messenger RNA (GFP-ASO) or a scrambled control oligo (Cntr-ASO) were delivered by gymnosis [1 μM] to HT1080, HeLa or HCT-116 cell lines. Two days later, the media was removed and, following washing, the cells were transfected with a 5′ end-capped, polyadenylated eGFP mRNA. eGFP fluorescence is visible in cells treated with the Cntr-ASO (left column), but is absent in cells treated with the anti-GFP-ASO (right column).

The results obtained in the RACE PCR assay demonstrated the existence of mRNA cleavage products and did not support the possibility that the PSL-ASOs could function as steric blockers. To validate these findings, we performed a parallel analysis of the mRNA by quantitative real time PCR. Cellular levels of the targeted mRNA were reduced in synchrony with protein expression after gymnotic delivery of the PSL-ASO (Supplementary Figure S4). Furthermore, to confirm that mRNA targeting was occurring in the cell cytoplasm, we gymnotically delivered an anti-eGFP PSL-ASO (GFP-ASO) or a control, scrambled PSL-ASO (Cntr-ASO) to HT1080, HeLa and HCT-116 cells (Figure 7). Two days later, we transfected each of these cell lines with a 5′ end-capped, 3′-polyadenylated eGFP mRNA (eGFP mRNA). The mRNA is rapidly bound by the ribosome and translated to eGFP protein, which is detectable within a few hours (Supplementary Figure S7 shows the 5hr time point). In cells treated with the control PSL-ASO, eGFP fluorescence was readily detectable (Figure 7, Cntr-ASO). However, eGFP was almost completely absent in cells pre-treated with the GFP-ASO targeted to the eGFP mRNA (Figure 7, GFP-ASO).

To examine the possibility that a cellular relocation of the transfected eGFP mRNA could be due to the lipofection process and/or the process of cell division, we delivered the Cy5 eGFP- mRNA and observed its cellular distribution 24 h following transfection (Supplementary Figure S6). The mRNA remained cytoplasmic, and was localized mostly in the perinuclear region in a pattern resembling that of the cellular distribution of the PSL-ASO (Supplementary Figure S6) and of a transfected siRNA (Supplementary Figure S1). However, a very small quantity of Cy5 signal was detectable in the nuclear compartment of some cells. We cannot distinguish if this is due to free label entering the nucleus, or a small amount of RNA perhaps gaining access to this compartment in dividing cells.

To monitor the fate of the mRNA and protein expression levels when a PSL-ASO is delivered via gymnosis or transfected into cells, we delivered the Cy5 eGFP-mRNA in cells pre-treated with 1 μM or transfected with 30 nM of the GFP-PSL-ASO and included an anti-eGFP siRNA for comparison purposes (Supplementary Figure S7). We then monitored mRNA and eGFP protein expression starting 5 h after transfection. Our results suggest that regardless of the method of delivery (lipofection or gymnosis) or whether or not a PSL-ASO or a siRNA is employed for silencing purposes, protein expression is readily and strongly suppressed as early as 5 h after treatment (Supplementary Figure S7). Remarkably, a brighter Cy5 signal appears in the experimental samples when compared to their non-targeting controls; this might indicate that a block of translation (Supplementary Figure S7) is occurring. Eventually, of course, the targeted mRNA is degraded (Supplementary Figure S8).

These experiments strongly suggest that cytoplasmic PSL-ASO-targeting can occur, and that nuclear localization of the ASO is not an absolute requirement for antisense activity. We wish to clearly stress that we are not claiming that ASO silencing does not occur in the nucleus. Rather, our results suggest it can also occur in the cytoplasm. To further confirm that gymnotically delivered ASOs are still active in the nucleus, we investigated the effectiveness of a gymnotically delivered splice switching oligonucleotide (SS-ASO) designed to induce skipping of an exon which disrupts the eGFP coding sequence in HeLa cells (30). The SS-ASO was still capable of inducing GFP expression, but to a much lesser extent then when delivered via lipid transfection (Supplementary Figure S5). When taken together, our experiments strongly suggest that ASO activity occurs in both the nucleus and the cytoplasm. This point is further elaborated in the Discussion.

## DISCUSSION

The activity of PSL-ASOs, similar to all other antisense oligos, can occur in the cell nucleus through an RNase H-dependent recognition and endonucleolytic cleavage mechanism. In this work, we found that PSL-ASOs can perform their silencing activity in the cell cytoplasm (Figure 7, and Supplementary Figures S7 and S8) in addition to the nucleus. For splice-switching oligos, nuclear activity is still detectable after gymnotic delivery (Supplementary Figure S5). This activity is not as efficacious, and is also slower to appear than when the splice-switching oligo is delivered via lipofection (Supplementary Figure S5).

It has been speculated that ≈5% of the total intracellular RNase HI is present in the cytoplasm (31). At this time, however, we do not know if RNase H is the mRNA-cleaving endonuclease in cytoplasmic PSL-ASO-directed antisense activity. Human RNase H usually preferentially cleaves mRNA between the 8th and 12th nucleotide counting from the 3′ terminus of a hybridized PSL-ASO (32), However, the RACE-PCR analysis of the mRNA cleavage products produced by PSL-ASOs delivered gymnotically demonstrated a cleavage pattern not typical of human RNase H (Figure 6). These data indicate that other GW-body endonucleases may be responsible for the cytoplasmic degradation of the mRNA, although we cannot exclude the possibility that the inclusion of LNA modifications in the PS-ASOs may have altered the RNase H cleavage pattern.

RNase H-independent oligos can inhibit translation (2) through steric blockade, though this mechanism reduces protein expression without triggering mRNA degradation. PSL-ASOs, on the other hand, elicit RNAse H activity. The results obtained with the RACE PCR assay demonstrate that the target mRNA is degraded. This is confirmed by qPCR analysis, which demonstrated that in all cases (except that of eGFP, for which qPCR was not performed and the mRNA level was roughly assessed by measuring the Cy5 signal), cellular levels of the targeted mRNAs were reduced. (In the eGFP experiments, we cannot exclude the remote possibility that at least a small amount of the eGFP mRNA translocates to the nucleus, where it is silenced. This could happen during mitosis, but mitosis occurs on too long a time scale for our experiments).

Our data suggest a mechanism that is possibly initiated by a steric blockade of translation, which ultimately leads to the degradation of the target mRNA (Supplementary Figures S7 and S8). We postulate that this could be a consequence of the segregation of translationally blocked mRNAs into cytoplasmic bodies, similar to what occurs during microRNA gene regulation (33–36). Degradation of the mRNA then follows (Supplementary Figure S8).

Unexpectedly, we observed the same sequence of events (i.e. first possible stabilization, then degradation) when the oligo was either co-transfected (not shown) or sequentially transfected with the mRNA (Supplementary Figures S7 and S8). A similar, though not as dramatic a phenomenon was observed when a siRNA was employed instead of an oligo (Supplementary Figure S7).

It is conceivable that after transiting the late endosomal membrane by an unknown mechanism, the PSL-ASO/Ago-2 complex binds to its target mRNA, blocks translation and prompts formation of GW and p-bodies. The binding of PSL-ASOs to Ago-2 is possibly driven by the negative charge of the oligo, regardless of its sequence. It is also noteworthy that PSL-ASOs co-immuno-precipitate with the TCP-1 complex (19). TCP-1 forms a stable complex with Hsc-70 (37), which in turn has been implicated in the mechanism of endocytosis (38) and in the loading of siRNA into the PAZ domain of Ago-2 (39). Hsc-70 was also one of the proteins we identified through the mass spectrometry analysis of the immune-precipitates formed after the interaction of a PS-ASO and Ago-2 (not shown). Once the ASO is transferred into GW-bodies, the target mRNA may proceed into p-bodies as the final degradation locus.

Our results suggest that localization to late endosomes and binding to Ago-2 are events that enable PSL-ASO antisense activity. Here, however, Ago-2 does not exert endonucleolytic cleavage activity but rather seems to function more as an escort protein. We cannot entirely exclude an indirect effect of Ago-2 silencing, perhaps due to its downstream regulation of some other, heretofore unknown factor involved in ASO function. Ago-2 could also be part of a sorting mechanism that screens the content of late endosomes. The PSL-ASO/Ago-2 complex could subsequently proceed into the nucleus, depending perhaps on additional interactions with such proteins as Ago-1 and nucleolin.

We wish to stress that, while antisense oligos silencing does occur in the nucleus as demonstrated previously and, as our data strongly suggest, in the cytoplasm too, it is possible that under some circumstances, both pathways operate simultaneously. If the specific RNA species to be silenced resides solely in the nucleus, then oligo activity will of course occur in that location only. However, if the mRNA (or pre-mRNA) can potentially localize in both the cytoplasm and nucleus, respectively, the pathway that would be most prevalent/potent may be based on oligo chemistry and concentration, treatment time, cell type and the location and nature of the target.

Therefore, in conclusion, it appears to us that the mechanism of intracellular oligo silencing of at least some mRNA species may be more complicated than has been previously appreciated. Why should it be otherwise? PS oligos are highly complex molecules that can interact with a large number of intracellular proteins. The earliest studies on many drugs, not only on PS oligos, have proposed mechanisms of action that have been found not to be incorrect, or lacking, but rather, limiting; these mechanisms are often subsequently refined as data accrue through the performance of additional studies. Nevertheless, any assessment of mechanism is liable to the inherent limitations of microscopy as a tool for the definitive tracking of oligo localization, though such difficulties are ameliorated by the use of live-cell experiments. Further, there may be profound, heretofore unknown, implications that are contingent upon the dependence of Ago-2 for antisense oligo activity. It is our great hope that our results will encourage deeper examinations of these critical mechanistic issues and further exploration of our hypotheses by the scientific community.

## Supplementary Material

SUPPLEMENTARY DATA
