# Supplementary material for: A cytoplasmic pathway for gapmer antisense oligonucleotide-mediated gene silencing in mammalian cells
Source: Nucleic Acids Res. 2015 Oct 3;43(19):9350–61. doi: 10.1093/nar/gkv964 (PMC4627093; doi:10.1093/nar/gkv964)
Supplement: SUPPLEMENTARY DATA [file supp_43_19_9350__index.html]

A cytoplasmic pathway for gapmer antisense oligonucleotide-mediated gene silencing in mammalian cells — SUPPLEMENTARY DATA 

# A cytoplasmic pathway for gapmer antisense oligonucleotide-mediated gene silencing in mammalian cells

## SUPPLEMENTARY DATA

- SUPPLEMENTARY DATA
