# Supplementary material for: A cytoplasmic pathway for gapmer antisense oligonucleotide-mediated gene silencing in mammalian cells
Source: Nucleic Acids Res. 2015 Oct 3;43(19):9350–61. doi: 10.1093/nar/gkv964 (PMC4627093; doi:10.1093/nar/gkv964)
Supplement: SUPPLEMENTARY DATA [file supp_gkv964_nar-03391-y-2014-File009.doc]

**SUPPLEMENTARY DATA**

**A Cytoplasmic Pathway for Gapmer Antisense Oligonucleotide-Mediated Gene Silencing in Mammalian Cells**

Daniela Castanotto, Min Lin, Claudia Kowolik, LiAnn Wang, Xiao-Qin Ren, Harris S. Soifer, Troels Koch, Bo Rode Hansen, Brian Armstrong, Zhigang Wang, Paul Bauer, John Rossi and CA Stein.


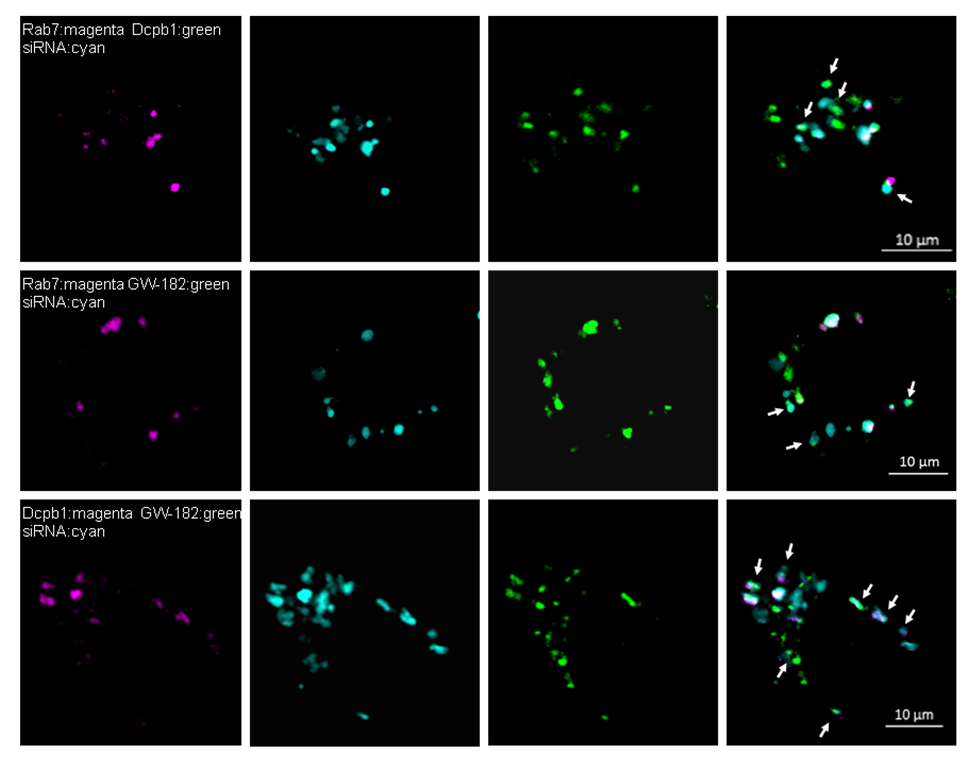


**Figure S1.** **siRNA distribution in degradation bodies.** The top row demonstrates partial siRNA (cyan) co-localization with Rab7 (magenta) and dcpb1 (green). In the upper right panel of the top row, co-localization of the siRNA to the late endosomes appears as a pink-white color and co-localization of the siRNA to p-bodies is indicated by arrows. The second row demonstrates partial co-localization of the siRNA with Rab7 (magenta) and to the GW-182 bodies (green). The latter is indicated by arrows. The third row demonstrates co-localization of the siRNA with p-bodies and with GW-182 bodies (indicated by arrows). The dcpb1 marker for p-bodies is shown in red, the GW-182 bodies in green and the siRNA in cyan.

**Figure S2. Ago-2 binds PSL-ASOs in HT1080 cells.** Immuno-precipitation (IP) of Argonaute complexes followed by nucleic acid recovery and gel analysis. A P^32^-labeled probe fully complementary to the PSL-ASO sequence (as described in the Experimental Procedures) was used to detect its presence in the immuno-precipitates. The U6 small nucleolar RNA was employed as a loading control.


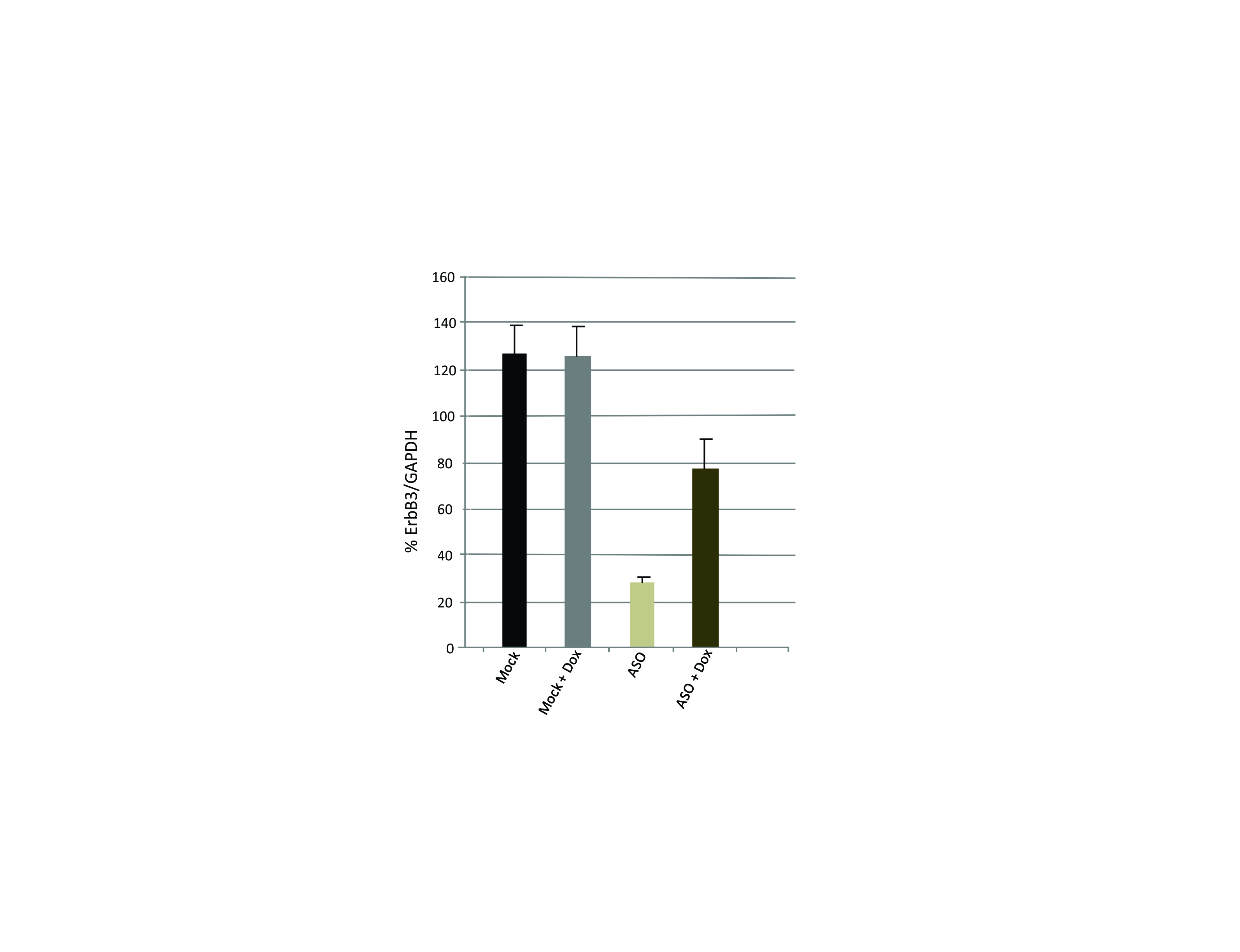


**Figure S3. Real time PCR demonstrates reduced PSL-ASO silencing of ErbB3 mRNA in a Ago-2 knockdown cell line.** A tetracycline (Tet) inducible stable cell line expressing an anti-Ago-2 shRNA (REF) or its Mock control (the parental 293T-Rex line) were seeded into 6-well plates and treated with 5 μg/ml of Doxycycline (Dox). 48 hours after treatment, 2.5 μM of an ErbB3-ASO was added to the media. Cells were collected after a further 48 hours. QPCR was performed as described in Material and Methods. A reduction in ASO activity after targeting the ErbB3 mRNA in cells treated with doxycycline (Dox) which expressed the anti-Ago-2 shRNA (ASO + Dox) was observed when compared to the absence of Dox (ASO). Doxycycline treatment did not affect ErbB3 mRNA levels in the control, parental line (Mock vs. Mock + Dox). The graph represents two biological replicates with triplicate experiments performed for each treatment.


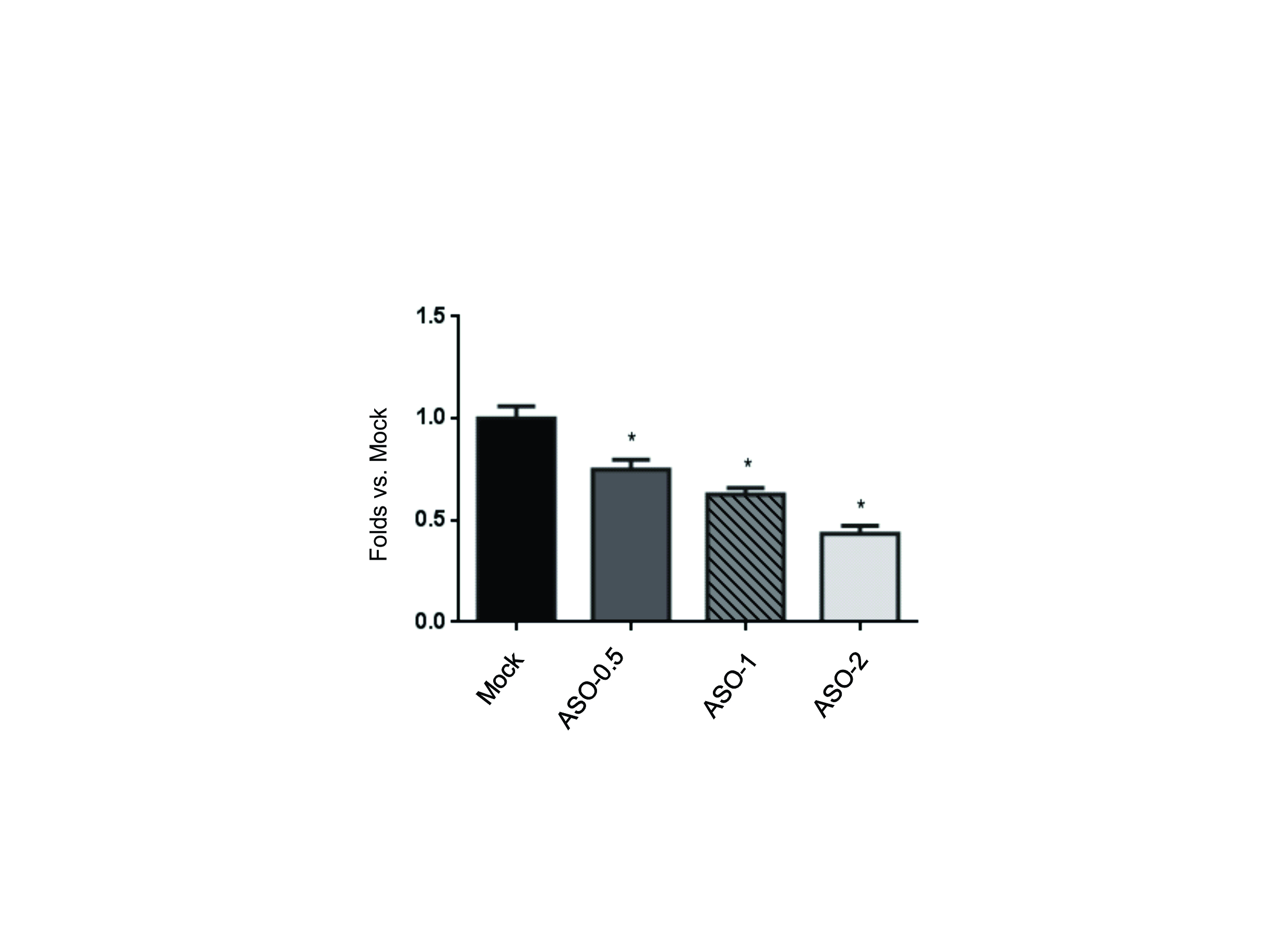


**Figure S4.**  **Real time PCR demonstrates PSL-ASO silencing of Bcl-2 mRNA expression** QPCR experiments show reduction of mRNA levels in cells treated with different concentrations [0.5μM, 1 μM and 2 μM] of the anti-Bcl-2 PSL-ASO (ASO-0.5, ASO-1 and ASO-2). Cells treated with a scrambled PSL-ASO at a concentration of 2 μM served as the Mock, which was used as the normalization control for these experiments. The graph represents the combined analysis of three different experiments including three technical replicates. p (Student’s t test) < 0.002.


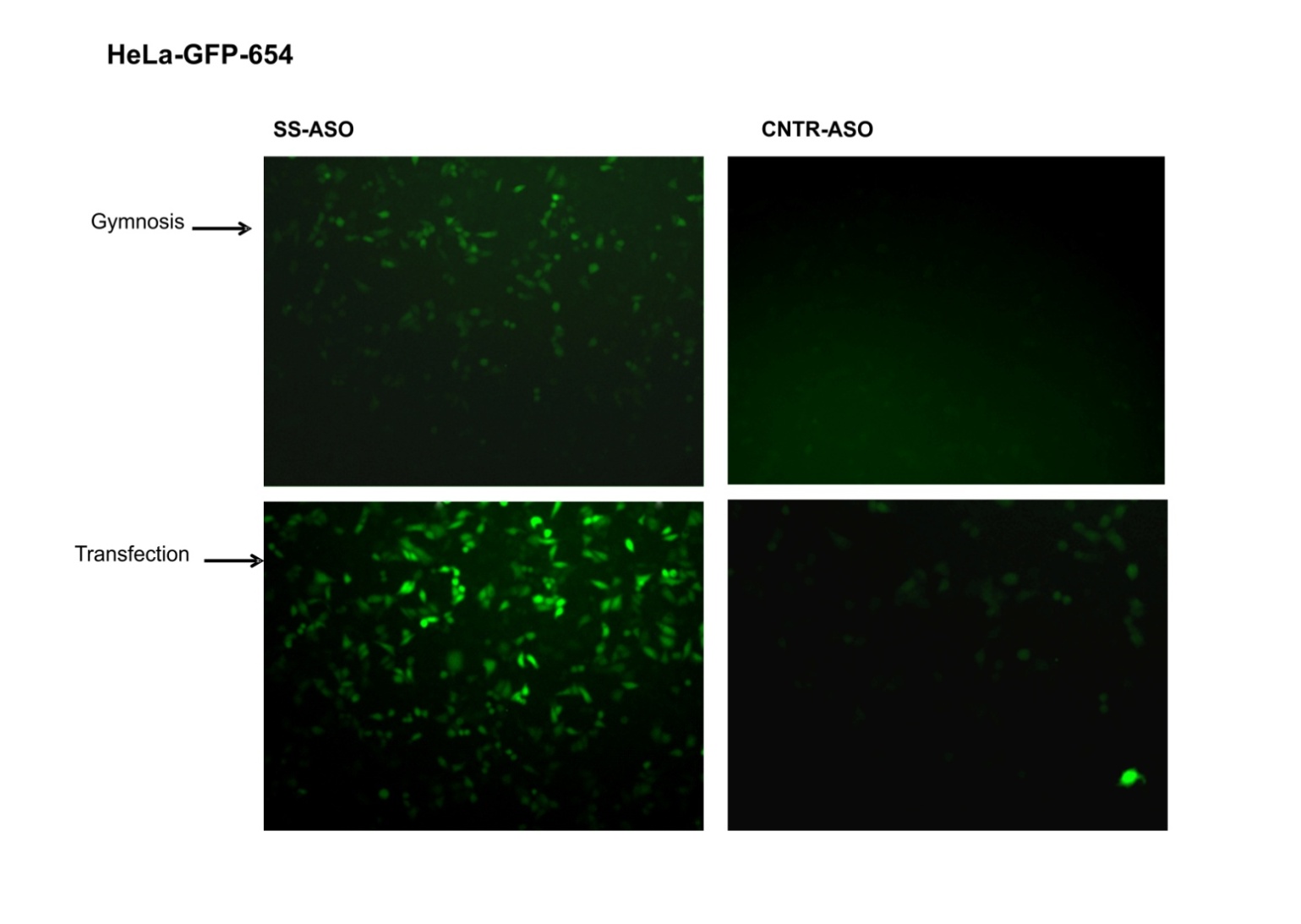


**Figure S5.**  **Gymnotically delivered splicing switch (SS) ASOs are active in the nucleus**  A SS-ASO [1 μM], designed to induce exon skipping and restore the eGFP coding sequence, was gymnotically delivered to HeLa 654 cells . eGFP expression is directly proportional to, and an indication of SS-ASO activity in the nucleus (top). A 50 nM concentration of this same SS-ASO was delivered via lipid transfection into the HeLa 654 cells (bottom). Images were taken 12 hrs after ASO transfection and 48 hrs after gymnotic ASO delivery. No detectable eGFP expression was observed 12-24 hours after gymnotic delivery.


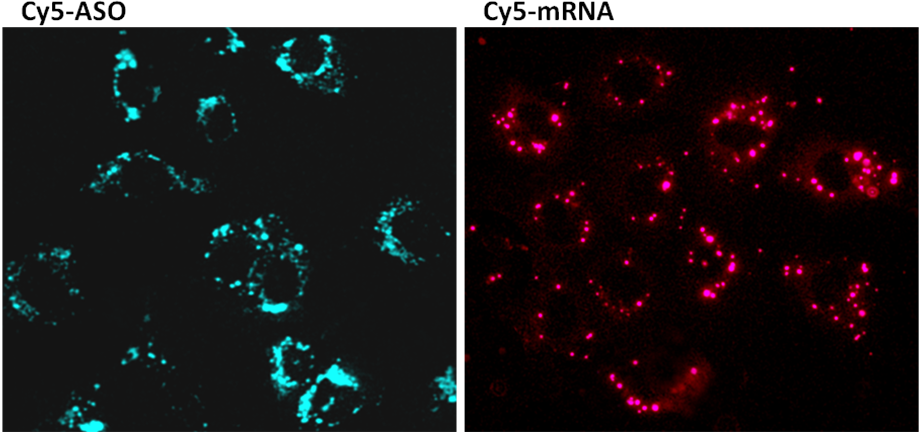
**Figure S6.**  **Lipofected mRNA** **preferentially localize to the perinuclear region of the cytoplasm.** The transfected Cy5-labeled, 5’-capped, 3’-polyadenylated eGFP mRNA (Cy5-mRNA, TriLink Bio Technologies) demonstrates mostly perinuclear accumulation of the fluorescent signal. Confocal Z-section imaging of a Cy5-labeled anti Bcl-2 PSL-ASO (Cy5-ASO) shows that both the gymnotically delivered Cy5-ASO and the lipofected Cy5-mRNA localize with a similar cytoplasmic pattern.


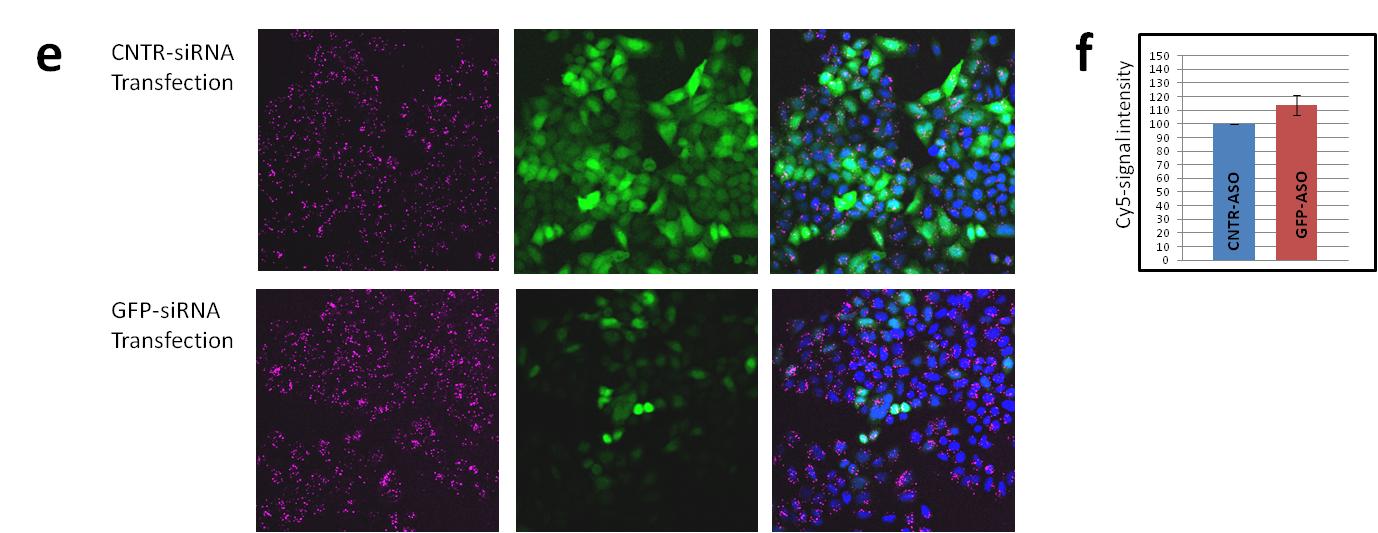

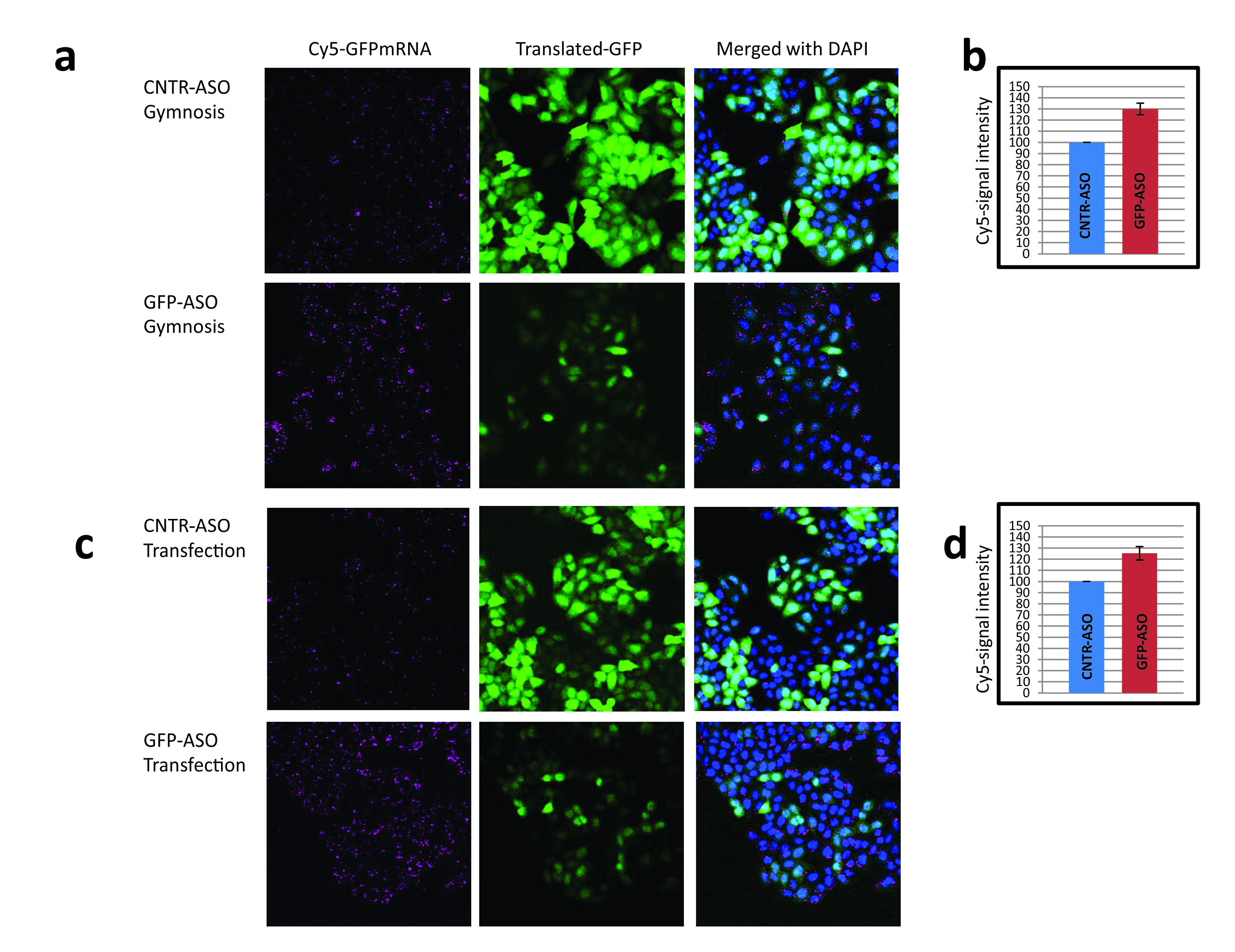


**Figure S7. A block of translation appears to be the very first observable silencing event subsequent to the binding of the targeting oligo.** A brighter Cy5 signal in the experimental samples (GFP-ASO) when compared to their non-targeting controls (CNTR-ASO), when there is relatively little eGFP visible, indicates that the mRNA is stabilized but may be translationally blocked (compare the first and the second columns, all rows). An oligo targeted against the Cy5-eGFP messenger RNA (GFP-ASO) or a scrambled control oligo (CNTR-ASO) were delivered by gymnosis **(a)** [1 μM] to HeLa cells. 24-hours later the same oligos **(c)** were lipofected into cells in other wells [30 nM]. After an additional 24-hours, the cells were transfected with a Cy5-labeled 5’end-capped, polyadenylated eGFP mRNA.  eGFP fluorescence is visible in cells treated with the Cntr-ASO, but is greatly diminished in cells treated with the anti-GFP-ASO (center column). The Cy5-mRNA signal (magenta, left column) is brighter in cells treated with the targeting, antisense oligo (compare the Cy5 signals in the first and second rows and in the third and fourth rows). A similar, though not as striking outcome was observed when a siRNA targeted against the eGFP messenger RNA (GFP-siRNA) or a control non-targeting siRNA (CNTR-siRNA) were lipofected [30 nM] into HeLa cells instead of the antisense oligos **(e)**. The right column for all rows shows the merged images, with the nuclei stained Hoechst 33342, which was added at 1 μg/ml for 5 minutes at 37 ^o^C. The images were taken 5 hours after Cy5-mRNA delivery. The graphs represent the combined analysis of three different experiments including two technical replicates. The increase in Cy5 fluorescent signal for the targeting oligos vs. the non-targeting oligos, which was quantified over the entire surface of wells containing equivalent number of cells, was approximately 30% for the gymnotically delivered targeting oligos **(b)**; 25% for lipofected targeting oligos **(d)**; and 14% for the lipofected targeting siRNAs **(f)**.


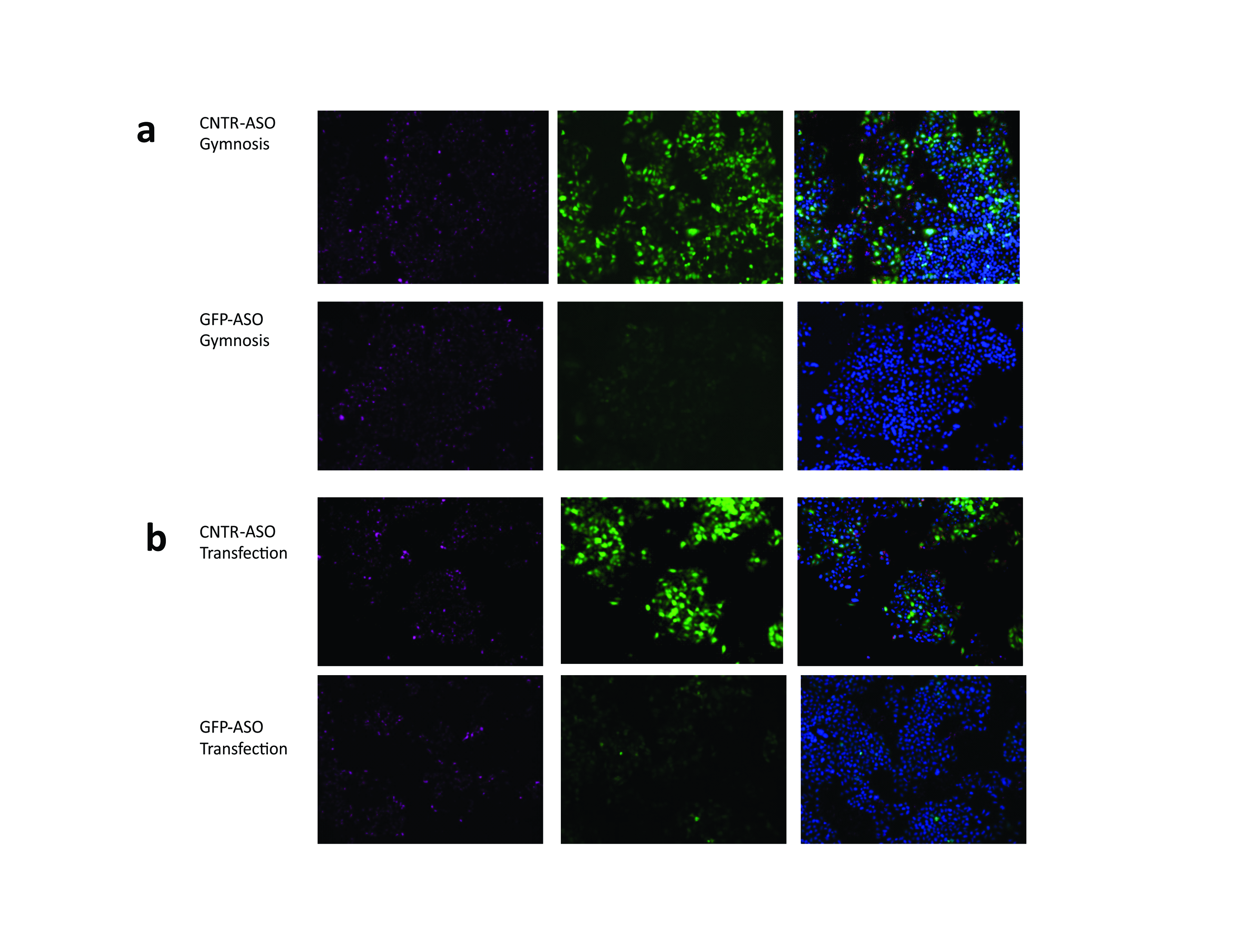


**Figure S8.**  **Targeted** **Cy5-mRNA is subject to time-dependent degradation.** An oligo targeted against the Cy5-eGFP messenger RNA (GFP-ASO) or a scrambled control oligo (CNTR-ASO) were delivered by gymnosis **(a)** [1 μM] to HeLa cells. 24-hours later the same oligos were lipofected **(b)** into other wells [30 nM]. After an additional 24-hours, the cells were transfected with a Cy5-labeled, 5’end-capped, polyadenylated eGFP mRNA.  eGFP fluorescence is visible in cells treated with the Cntr-ASO, but is absent in cells treated with the anti-GFP-ASO (center column). The Cy5-mRNA signal (magenta, first column) was reduced when compared to the 5-hour time point in Figure 8. The last column for both rows depicts the merged images. For nuclear staining, Hoechst 33342 was added at 1 μg/ml for 5 minutes at 37 ^o^C. The images were taken 48 hours after the Cy5-mRNA delivery.
